# Continuous-Infusion Vancomycin in Neonates: Assessment of a Dosing Regimen and Therapeutic Proposal

**DOI:** 10.3389/fped.2019.00188

**Published:** 2019-05-14

**Authors:** Manon Tauzin, Robert Cohen, Xavier Durrmeyer, Gilles Dassieu, Jérôme Barre, Laurence Caeymaex

**Affiliations:** ^1^Neonatal Intensive Care Unit, Centre Hospitalier Intercommunal de Créteil, Créteil, France; ^2^ACTIV, Association Clinique et Thérapeutique Infantile du Val de Marne, Saint-Maur des Fossés, France; ^3^Université Paris Est, IMRB- GRC GEMINI, Créteil, France; ^4^Clinical Research Center, Centre Hospitalier Intercommunal de Créteil, Créteil, France; ^5^Groupe de Pathologie Infectieuse Pédiatrique, Paris, France; ^6^Unité Court Séjour, Petits Nourrissons, Service de Néonatologie, Centre Hospitalier Intercommunal de Créteil, Créteil, France; ^7^Inserm, U1153, Obstetrical, Perinatal and Pediatric Epidemiology Team, Epidemiology and Biostatistics Sorbonne, Paris Descartes University, Paris, France; ^8^Department of Pharmacology, Centre Hospitalier Intercommunal de Créteil, Créteil, France; ^9^Department of Research in Ethics EA1610 Studies on Science and Technics, Paris Est University, Créteil, France

**Keywords:** vancomycin, continuous-infusion, neonates, dosing, pharmacokinetics

## Abstract

**Introduction:** Vancomycin remains the reference antibiotic in neonates for care-related infections caused by ß-lactam–resistant Gram-positive bacteria. Achieving the optimal serum vancomycin level is challenging because of high inter-individual variability and the drug's narrow therapeutic window. Continuous infusion might offer pharmacokinetic and practical advantages, but we lack consensus on the dosing regimen. The aim was to determine the proportion of neonates achieving an optimal therapeutic vancomycin level at the first vancomycin concentration assay and which dosing regimen is the most suitable for neonates.

**Methods:** All neonates receiving continuous-infusion vancomycin (loading dose 15 mg/kg and maintenance dose 30 mg/kg/d) in a neonatal intensive care unit were retrospectively analyzed. The proportion of neonates reaching the target serum vancomycin level was calculated. After reviewing the literature to identify all published articles proposing a dosing regimen for continuous-infusion vancomycin for neonates, regimens were theoretically applied to our population by using maintenance doses according to covariate(s) proposed in the original publication.

**Results:** Between January 2013 and December 2014, 75 neonates received 91 vancomycin courses by continuous infusion. Median gestational age, birth weight, and postnatal age were 27 weeks (interquartile range 26–30.5), 815 g (685–1,240), and 15 days (9–33). At the first assay, only 28/91 (30.8%) courses resulted in vancomycin levels between 20 and 30 mg/L (target level), 23/91 (25.3%) >30 mg/L and 40/91 (43.9%) <20 mg/L. We applied six published dosing regimens to our patients. One of these dosing regimens based on corrected gestational age (CGA) and serum creatinine level (SCR) would have allowed us to prescribe lower doses to neonates with high vancomycin levels and higher doses to neonates with low levels.

**Conclusions:** A simplified dosing regimen of continuous-infusion vancomycin did not achieve therapeutic ranges in neonates; a patient-tailored dosing regimen taking into account CGA and SCR level or an individualized pharmacokinetic model can help to anticipate the inter-individual variability in neonates and would have been more suitable.

## Introduction

Vancomycin is a glycopeptide antibiotic frequently prescribed for neonatal care-related infections caused by Gram-positive bacteria such as coagulase-negative Staphylococci (CoNS), methicillin-resistant *Staphylococcus aureus* (MRSA), and Enterococci species (1). Because of the narrow therapeutic window and the lack of consensus on a toxicity threshold, determining the optimal dosing regimen for vancomycin is difficult, particularly for premature neonates, including those born before 28 weeks' gestation.

Vancomycin is a time-dependant antibiotic. It is sensitive to high inoculum effect and inhibited by biofilms (2). Its pharmacokinetics are characterized by an unbound fraction higher in neonates (about 90%) than adults and children (3), a nearly total renal clearance correlated with creatinine clearance and a half life of 3.5 to 10 h (2, 4). Vancomycin is administered intravenously because of its very low oral bioavailability. At all ages and even more so in neonates, the serum levels of vancomycin achieved feature high inter-individual variability (4). Covariates reported to influence its clearance are weight, gestational age (GA), corrected gestational age (CGA), post-natal age (PNA), co-medication with non-steroidal anti-inflammatory drugs, and serum creatinine (SCR) level, which could be interrelated (4, 5).

A ratio of 24-h area under the curve (AUC_0−24h_) to minimum inhibitory concentration (MIC) >400 mg.h/L for total vancomycin is considered predictive of optimal antibacterial efficiency against MRSA in adults (6). This AUC_0−24h_/MIC ratio is based on studies of adults using discontinuous infusion of vancomycin with a target trough level of 15 to 20 mg/L (7). In clinical practice, the AUC_0−24h_/MIC ratio is difficult to estimate during discontinuous infusion (8) but is easy to estimate with steady-state levels during continuous infusion with the assumption that the level is relatively stable during infusion. For instance, for *S. aureus* infection [modal MIC 1 mg/L, MIC breakpoint 2 mg/L (9)], a steady-state level of vancomycin ≥17 mg/L corresponds to an AUC_0−24h_/MIC ratio ≥400 mg.h/L. Concerning CoNS infection, consistent data for neonates in an experimental animal model suggested an optimal efficacy of vancomycin and a decrease in induced resistance with an AUC_0−24h_/MIC ratio >400 mg.h/L (10). These data have not been validated in human neonates. For CoNS [modal MIC 2 mg/L, MIC breakpoint 4 mg/L (9)], a steady-state level of 33 mg/L would be necessary to achieve an AUC_0−24h_/MIC ratio >400 mg.h/L.

Because of the lack of consensus on efficacy and toxicity thresholds and an optimal dosing regimen, vancomycin dosing regimens between centers exhibit large heterogeneity (11). Continuous administration is controversal, notably in neonates, and data are mostly extrapolated from adult studies (12). Continuous administration requires a continuous availability of line but neonates, especially preterm neonates, often have a central catheter. Furthermore, vancomycin is stable for at least 48 h and compatible with parenteral nutrition (13) and the only one practical limitation can be drug incompatibilities. However, the major advantage of continuous infusion in neonates concomitant to parenteral nutrition is practical: preventing numerous catheter manipulations could decrease the risk of catheter-related infection (14). Therapeutic levels are achieved faster with continuous infusion, without clinical superiority as compared with discontinuous infusion (15, 16). Furthermore, with continuous infusion, fewer blood samples are required to adjust the optimal dosing regimen, possibly because therapeutic levels are achieved faster (17, 18). A steady-state level can be sampled at any time, for example, concomitant to a scheduled blood test, and is easier to interpret than trough levels (17, 19).

Few data are available on continuous infusion in neonates, particularly for premature infants born before 28 weeks' gestation (5). Different dosing regimens and different target levels have been proposed; with this heterogeneity, choosing an optimal dosing strategy in neonatology is difficult. Pharmacokinetics studies tend to develop individualized pharmacokinetic models with patient-tailored dosing regimens (8, 20).

The aim of this study was to assess the efficiency of a simplified dosing regimen of continuous-infusion vancomycin (loading dose 15 mg/kg and maintenance dose 30 mg/kg/d) in a neonatal intensive care unit (NICU) in neonates with suspected care-related infections caused by ß-lactam–resistant Gram-positive bacteria. We assessed the proportion of neonates with serum vancomycin level achieving the chosen target with a first therapeutic drug monitoring. We then determined which dosing regimen, among those published, would have been the most adequate in our population.

## Material and Methods

### Population and Collected Data

We included all neonates receiving vancomycin with at least one serum vancomycin concentration assay between January 2013 and December 2014 in the NICU of Center Hospitalier Intercommunal de Créteil (France). The following data were retrospectively collected from patient charts: GA in weeks (wks) based on the best obstetrical estimate, birth weight (g), intra-uterine growth restriction (IUGR, birth weight <10th percentile for gestational age), sex, PNA in days (d), corrected gestational age (wks), current weight (g), SCR level (μmol/L) before treatment (quantified by the Jaffe method, Roche diagnostics, Indianapolis, IN, USA), serum vancomycin level (mg/L), delay between loading dose and blood sample (h), and microbiological data (results of blood cultures). No other consent than consent to usual standard care from parents was requested because the studied treatment was a standard of care. The local ethics committee of Créteil hospital approved the anonymous collection of data and their publication.

### Vancomycin Dosing Regimen

Vancomycin was administered by continuous infusion via a central or peripheral catheter starting with a loading dose of 15 mg/kg over 1 h followed by a maintenance dose of 30 mg/kg/d (21). A blood sample was drawn at least 18 h after the loading dose to reach the steady state (20). Serum vancomycin level was determined by the immunoturbidimetric technique (QMS system of Vancomycin, Seradyn Inc., Indianapolis, IN, USA). If the target level was not reached in the first sample, the maintenance dose was adjusted according to clinicians' choice and a new vancomycin assay was performed at least 24 h after dose adjustment until the target level of 20 to 30 mg/L was achieved.

### Assessed Outcomes

Our first goal was to determine the proportion of neonates reaching the target serum vancomycin level and to identify independent covariates associated with low or high therapeutic levels. The target level used was 20 to 30 mg/L (19).

Our second objective was to assess different continuous-infusion regimens proposed in the literature as applied to our population. We determined whether each proposed regimen would have resulted in a significantly higher, equal or lower vancomycin dose within each subgroup of our population: infants with a first therapeutic drug monitoring <20 mg/L (underdose), 20 to 30 mg/L (appropriate dose), and >30 mg/L (overdose). For each patient, we used the covariate(s) proposed in the original publication.

### Literature Search

MEDLINE was searched via PubMed to identify all published articles proposing a dosing regimen for continuous-infusion vancomycin for neonates. The search was conducted in August 2018 with the following terms: neonates AND vancomycin AND (dosing OR continuous). We selected English-language articles that proposed a detailed dosing regimen for continuous infusion of vancomycin in neonates that could be reproduced in our population.

### Statistical Analysis

The association between vancomycin levels and variables (GA, birth weight, PNA, CGA, current weight, IUGR, sex, timing of assay, and SCR level) was analyzed by using R 3.3.2 (http://www.R-project.org). To facilitate results' interpretation for clinicians, continuous variables were separated into dichotomous variables with cut-offs set according to the literature and commonly used categories: GA < or ≥ 28 weeks (22), CGA < or ≥ 32 weeks (22), neonate weight ≤ or > 1,000 g (23), PNA ≤ or > 14 days (24, 25), timing of assay ≤ 24 h or > 24 h (20), and SCR level < or ≥ 70 μmol/L (20, 21). Descriptive data are described with median and interquartile range (IQR) or mean (SD). Chi-square or Fisher exact test was used to determine covariates with significantly different distribution among vancomycin level subgroups (low level, target level, and high level). *P* ≤ 0.05 was considered statistically significant. Covariates with *p* < 0.1 on univariate analysis were included in a multivariate analysis. We used multinomial logistic regression to determine covariates independently related to low and high levels as compared with the target level (*p* < 0.05), estimating adjusted odds ratios (aOR) and 95% confidence intervals (CIs).

To compare doses proposed by each dosing regimen reported in the literature with actual doses given to our patients, we used Student *t*-test and report mean differences and 95% CIs.

## Results

### Population

From January 2013 to December 2014, 75 neonates received 99 vancomycin courses by continuous infusion. Eight treatments were excluded because the neonates did not receive the dosing regimen evaluated. Patient characteristics are summarized in Table 1. Median gestational age and birth weight were 27 weeks (IQR 26–30.5) and 815 g (IQR 685–1,240); 40/75 neonates (53.3%) were born before 28 weeks' GA. Overall, 184 serum vancomycin samples were assayed, with a median of 2 (IQR 1–2.5) determinations per patient. A first blood sample was taken 18.5 to 93.5 h after the loading dose. SCR level before treatment was available for 68 patients and ranged from 15 to 116 μmol/L; for 17/68 (25%) neonates, the SCR level was >70 μmol/L.

**Table 1:** Demographic and clinical characteristics of neonates (*n* = 75) under continuous infusion with vancomycin (*n* = 91 therapy episodes) for care-related infections caused by ß-lactam–resistant Gram-positive bacteria.

	***N* (%)**	**Median [IQR]**
Gestational age (weeks)		27 [26–30.5]
<28	40 (53.3)	
≥28	35 (46.7)	
Birth weight (g)		815 [685–1,240]
≤1,000	41 (54.7)	
>1,000	34 (45.3)	
Intrauterine growth restriction	33 (44)	
Sex		
Male	43 (57.3)	
Female	32 (42.7)	
Postnatal age (d)		15 [9–33]
≤14	42 (46.2)	
>14	49 (53.8)	
Corrected gestational age (weeks)		30 [28–34.5]
<32	52 (57.2)	
≥32	39 (42.8)	
Current weight (g)		1,230 [940–1,790]
≤1,000	30 (33)	
>1,000	61 (67)	
Serum creatinine level before treatment (μmol/L) (*n* = 68)		52 [26.5–70]
>70	17 (25)	
≤70	51 (75)	
First vancomycin level (mg/L)		21.1 [16.3–29.7]
Number of samples per treatment		2 [1–2.5]
Time of first sampling (h)		25.5 [23.9–33.3]
Treatment duration (d)		4 [3–7]

### Microbiological Data

For 24/91 (26.4%) courses, at least one blood culture was positive: 17/91 only one positive blood culture and 7/91 (7.7%) at least two positive blood cultures. Most (67/91; 73.6%) had negative blood cultures. Bacterial species found were CoNS (17/91; 18.7%), *S. aureus* (3/91; 3.3%) with one MRSA, *Streptococcus sp*. (2/91; 2.2%), *Escherichia coli* (1/91; 1%), and *Streptococcus pneumoniae* (1/91; 1.1%). The distribution of CoNS was *S. epidermidis* (*n* = 10), *S. haemoliticus* (*n* = 2), and *S. capitis* (*n* = 5).

### Serum Vancomycin Levels

**Figure 1:**
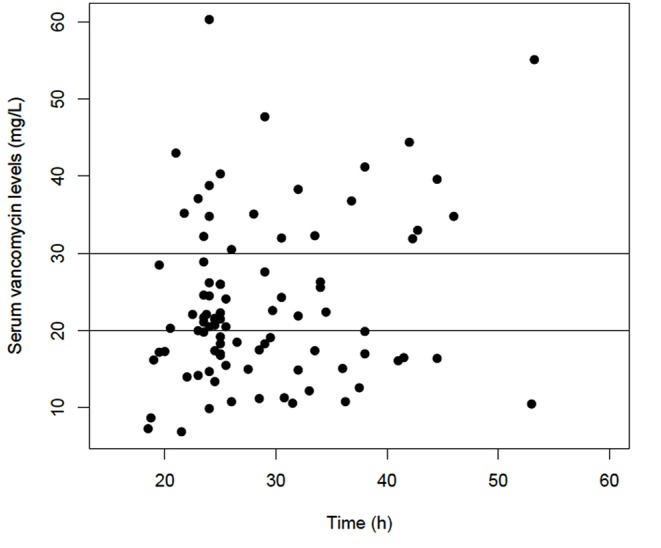
Serum vancomycin level (mg/L) on time (h). Horizontal lines represent the target level of 20 to 30 mg/L.

Vancomycin serum levels ranged from 6.9 to 60.3 mg/L. For 28/91 (30.8%) courses, a level of 20 to 30 mg/L was achieved in the first assay; 40/91 (43.9%) had low therapeutic levels, <20 mg/L, and 23/91 (25.3%) had levels >30 mg/L. For seven (7.7%), the level was >40 mg/L. Vancomycin levels on time are displayed in Figure 1. A total of 49 neonates had a second sample assayed and for 18/49 (36.7%), serum levels were at the chosen target; three retained a level >40 mg/L. At the first assay or after dose titration, for 56/91 (61.5%) courses, the therapeutic level between 20 and 30 mg/L was reached. For those neonates, the median time to achieving a therapeutic level was 34 h (IQR 24.5–74.5).

### Covariates Analysis

On univariate analysis, we identified five covariates significantly related to vancomycin serum levels: PNA (*p* = 0.001), sex (*p* = 0.05), CGA (*p* = 0.004), current weight (*p* = 0.04), and SCR level (*p* < 0.0001) (Table 2). On multivariate analysis, only three of these covariates remained significantly associated with serum vancomycin level: PNA > 14 days (aOR = 25.6, 95% CI [3.2–201.5], *p* = 0.002) and CGA ≥ 32 weeks were related to low level (aOR = 48.2, 95% CI [7.0–334.6], *p* < 0.001) and SCR level > 70 μmol/L was associated with high level (aOR = 33.0, 95% CI [4.0–272.6], *p* = 0.001). There was no association between timing of vancomycin assay and vancomycin serum levels (*p* = 0.4) (Table 2 and Figure 1).

**Table 2:** Distribution of covariates by vancomycin level.

**Covariates**	**Vancomycin level (mg/L)**			
	**<20**	**20–30**	**>30**	***p*-value**
Gestational age (wks)				
<28	19	17	16	0.16
≥28	21	12	6	
Birth weight (g)				
≤1,000	24	17	12	0.9
>1,000	16	12	10	
Intra-uterine growth restriction				
Absence	18	16	13	0.5
Presence	22	13	9	
Sex				
Male	18	16	17	0.05
Female	22	13	5	
Postnatal age (d)				
≤14	10	17	15	0.001
>14	30	12	7	
Corrected gestational age (wks)				
<32	21	21	15	0.004
≥32	8	8	25	
Current weight (g)				
≤1,000	8	11	11	0.04
>1,000	32	18	11	
Serum creatinine level (μmol/L)				
≤70	25	20	6	<0.0001
>70	2	3	12	
Time of first sampling (h)				0.4
≤24	11	12	7	
>24	29	16	15	

### Literature Search

The search algorithm identified 146 studies in Pubmed (144 after removal of duplicates): 51 referred to discontinuous infusion of vancomycin; 29 did not propose a detailed dosing schedule of vancomycin; 40 did not address our subject; 10 were not in English; six proposed a dosing schedule for neonates on extracorporeal life support or intraventricular injection; and two presented a preventive dosing regimen. Finally, six studies detailed different dosing regimens of continuous-infusion vancomycin for neonates (Table 3). Five studies agreed on a loading dose of 7 to 20 mg/kg to quickly reach target levels (15, 20, 21, 26, 28). Maintenance doses ranged from 10 to 60 mg/kg/d, with a unique dose of 30 mg/kg/d (21) or a dose adjusted to SCR level, CGA, birth weight, current body weight, and/or PNA (15, 20, 26–28). The proportion of patients achieving the chosen target level of 10 to 30 mg/L (21, 26), 10 to 25 mg/L (27) or 15 to 25 mg/L (15, 20) were 70.7% (20), 75% (27), 77% (15), 88% (26), 89.2% (21), respectively.

**Table 3:** Proposed dosing regimens from the literature.

**References**	**Population**	**Loading dose (mg/kg)**	**Maintenance dose (mg/kg/d)**	**Covariates**
Pawlotsky et al. (26)	Patients, 29 GA: 30.5 (3.7) weeks^a^ CGA: 33.9 (4.8) weeks^a^	7		CGA (wks)
			10	25–26
			12	27–28
			15	29–30
			18	31–32
			20	33–34
			23	35–36
			26	37–38
			29	39–40
			31	41–42
			34	43–44
			40	>45
Plan et al. (27)	Patients, 72 GA < 34 weeks	0	30	SCR level < 90 μmol/L
			20	SCR level > 90 μmol/L
Oudin et al. (21)	Patients, 47 GA 29 (23–41) weeks^b^ CGA 32.7 (27–47) weeks^b^	20	30	
Zhao et al. (20)	Patients, 116 CGA 33.8 (5.3) weeks^a^	= Target concentration × Vd (in mg)	= Target concentration × CL × 24 h (in mg/24 h)	Vd = 0.791 × (cW/1,416)^0.898^CL = 0.0571 × (cW/1,416)^0.513^ x (bW/1,010)^0.599^ × (1+0.282 × (PNA/17)) × (1/(SCR/42)^0.525^)
Patel et al. (15)	Patients, 60 GA 29 (24–41) weeks^b^ CGA 36 (26–62) weeks^b^	15	50	SCR level < 40 μmol/L and CGA > 40 weeks
			40	SCR level < 40 μmol/L and CGA < 40 weeks
			30	SCR level 40–60 μmol/L
			20	SCR level > 60 μmol/L
Janssen et al. (28)	Patients, 464 GA 32 (24–41) weeks^b^ CGA 34 (25–44) weeks^b^	10.5 to 13	25	PNA ≤ 7 days + bW ≤ 1,000 g
				PNA 8–14 days + bW ≤ 700 g
			27	PNA ≤ 7 days + bW 1,000–1,500 g
			30	PNA ≤ 7 days + bW 1,500–2,500 g
				PNA 14–28 days + bW ≤ 700 g
			32	PNA 8–14 days + bW 700–1,000 g
				PNA >28 days + cW < 2.5 kg
			36	PNA ≤ 7 days + bW > 2,500 g
				PNA 8–14 days + bW 1,000–1,500 g
			40	PNA 8–14 days + bW 1,500–2,500 g
				PNA > 28 days + cW 2.5–5 kg
			42	PNA 14–28 days + bW 700–1,000 g
			45	PNA 14–28 days + bW 1,000–1,500 g
			48	PNA 8–14 days + bW > 2,500 g
			52	PNA 14–28 d + bW 1,500–2,500 g
				PNA >28 days + cW 5–10 kg
			60	PNA 14–28 days + bW > 2,500 g
				PNA > 28 days + cW > 10 kg

GA, gestational age; CGA, corrected gestational age; SCR, serum creatinine; Vd, volume of distribution; CL, clearance; cW, current weight; bW, birth weight; PNA, post natal age.

a Mean (SD).

b Median (range).

### Dosing Regimen From the Literature Applied to Our Population

We compared mean doses and mean differences between our maintenance dose (30 mg/kg/d) and maintenance dose proposed by each dosing regimen from the literature (Table 4).

**Table 4:** Mean doses and mean differences (95% CIs) proposed by the six dosing regimens evaluated for each group of patients (low, target and high vancomycin levels).

**Dosing regimen**		**Low level** **(<20 mg/L)** **(*n* = 40)**	**Target level** **(20–30 mg/L)** **(*n* = 28)**	**High level** **(>30 mg/L)** **(*n* = 23)**
Our dosing regimen	Mean dose (SD)	30 (0)	30 (0)	30 (0)
	Mean difference (95% CI)	–	–	–
	*P*-value^a^	–	–	–
Pawlotsky et al.(26)	Mean dose (SD)	20.6 (6.5)	16.1 (4.6)	15.7 (6.8)
	Mean difference (95% CI)	−9.4 (−11.5 to −7.4)	−13.9 (−15.6 to −12.1)	−14.3 (−11.4 to −17.2)
	*P*-value^a^	<0.001	<0.001	<0.001
Plan et al. (27)	Mean dose (SD)	30 (0)	30 (0)	24.4 (5.1)
	Mean difference (95% CI)	0	0	−5.6 (−8.3 to −2.9)
	*P*-value^a^	–	–	<0.001
Oudin et al. (21)	Mean dose (SD)	30 (0)	30 (0)	30 (0)
	Mean difference (95% CI)	0	0	0
	*P*-value^a^	–	–	–
Zhao et al. (20)	Mean dose (SD)	48.5 (20)	30.8 (8.1)	25.4 (12.8)
	Mean difference (95% CI)	18.5 (10.7 to 26.4)	0.8 (−2.8 to 4.4)	−4.6 (−10.7 to 1.5)
	*P*-value^a^	<0.001	0.63	0.13
Patel et al. (15)	Mean dose (SD)	38.5 (9.5)	31.4 (8.3)	22.6 (7.3)
	Mean difference (95% CI)	8.5 (4.8 to 12.3)	1.4 (−2.3 to 5.1)	−7.4 (−10.9 to −3.8)
	*P*-value^a^	<0.001	0.45	<0.001
Janssen et al. (28)	Mean dose (SD)	36.4 (8.1)	32.2 (5.6)	33.8 (6.4)
	Mean difference (95% CI)	6.4 (3.8 to 9.0)	2.2 (0.1 to 4.4)	3.8 (1.0 to 6.6)
	*P*-value^a^	<0.001	0.04	0.009

a Student t-test comparing mean dose of our dosing algorithm with mean dose of each dosing regimen.

*95% CI, 95% confidence interval*.

Three dosing regimen from the literature would have proposed significantly higher vancomycin doses to neonates with a low vancomycin level, <20 mg/L: mean dose (SD) of 36.4 (8.1) mg/kg/d (27), 38.5 (9.5) mg/kg/d (15), and 48.5 (20) mg/kg/d (20). Two dosing regimens would have proposed the same dose: mean dose (SD) of 30 (0) mg/kg/d (21, 27). One dosing regimen would have proposed lower doses to neonates with low vancomycin level: mean dose (SD) of 20.6 (6.5) mg/kg/d (26) (Table 4).

For patients with a vancomycin level in the target range (20–30 mg/L), four dosing regimens would have proposed similar doses as the actual dose given to our patients: mean dose (SD) of 30 (0) (21, 27), 30.8 (8.1) (20), and 31.4 (8.3) (15) mg/kg/d. One would have proposed significantly lower doses: mean dose (SD) of 16.1 (4.6) mg/kg/d (26) and one significantly higher doses: mean dose (SD) of 32.2 (5.6) mg/kg/d (28) (Table 4).

For patients with a high vancomycin level, >30 mg/L, three dosing regimens would have proposed significantly lower doses: mean dose (SD) of 22.6 (7.3) (15), 15.7 (6.8) (26), and 24.4 (5.1) (27) mg/kg/d. Two would have proposed similar doses [25.4 (12.8) (20), and 30 (0) mg/kg/d (21)] and one significantly higher doses [33.8 (6.4) mg/kg/d (28)] (Table 4).

Finally, one dosing algorithm (15) would have proposed lower doses to patients with high levels, similar doses to those with levels in the target range and higher doses to those with low levels (Table 4).

## Discussion

This study presents a cohort of premature neonates receiving continuous infusion of vancomycin and is the first study comparing the six dosing regimens reported in the literature for neonates. We evaluated the proportion of patients reaching the therapeutic vancomycin range. With the dosing regimen used in our department (loading dose of 15 mg/kg and maintenance dose of 30 mg/kg/d), only 30.8% of neonates achieved target vancomycin levels of 20 to 30 mg/L, 43.9% had low therapeutic levels and 25.3% had levels >30 mg/L. These disappointing results confirm that a simplified regimen does not fit a population of neonates characterized by high inter-individual variability and the important pharmacokinetic changes in the first weeks of life (kidney maturation and changes in volume of distribution) (4).

After comparing dosing regimens proposed in the literature, the dosing regimen proposed by Patel et al. seemed the most adequate for our population. This model is easy to apply and includes cofactors of variability such as SCR level and CGA. Applied to our neonates with a target level of 20 to 30 mg/L, the model proposed significantly lower doses to neonates with high vancomycin levels and higher doses to those with low therapeutic levels. The individualized pharmacokinetic model proposed by Zhao et al. would also propose more appropriate doses to a large proportion of neonates. Indeed, it proposed significantly higher doses to neonates with low levels and lower doses to those with high levels (even if not significant, *p* = 0.13). This model can be adjusted to the chosen target level, which is an advantage when the clinician wants to adjust the target level to the MIC. In a previous study, a prospective validation of this model with 190 neonates showed that 72% reached the target level (15–25 mg/L) at the first therapeutic drug monitoring (29). The other evaluated dosing regimens proposed lower doses, even for patients with already low therapeutic levels (26), or higher doses, even for patients with already high levels (28). Those dosing regimen do not fit our population. An explanation could be the differences between vancomycin and serum creatinine measurement methods as demonstrated in some studies (30).

Concerns have been raised about the efficacy threshold of target concentrations because of increased vancomycin MICs with CoNS infection (4) and toxicity issues. According to the European Committee on Antimicrobial Susceptibility Testing (9), the modal MIC is 2 mg/L for CoNS and 1 mg/L for *S. aureus* infections. To reach the AUC_0−24h_/MIC ratio >400 mg.h/L as advised, a steady-state level >33 mg/L for CoNS infection and >17 mg/L for *S. aureus* infection would be needed to reach optimal efficiency and limit resistance (2, 18, 21). *S. aureus* infections are characterized by severe clinical presentation and metastatic risk (31). CoNS infections, with central line catheters, are rarely metastatic and have less severe clinical and biological presentations, but therapeutic failure can be attributed in part to biofilms produced (32). Moreover, the AUC_0−24h_/MIC target >400 mg.h/L was developed for MRSA infections, so a CoNS-specific pharmacodynamic target should be established, especially for neonates (33). Hence, for CoNS, an initial level < 30 mg/L could be sufficient despite a high MIC.

Concerning the upper threshold of the target concentration, a study of adults showed an AUC_0−24h_ >1,300 mg.h/L, corresponding to a steady-state level >55 mg/L, associated with increased risk of nephrotoxicity (34). Adult and pediatric studies suggest that without reaching such high levels, an upper threshold might be about 30 mg/L (35) (AUC_0−24h_ 700 mg.h/L), which might be increased to 37.5 mg/L (AUC_0−24h_ 900 mg.h/L), an adequate threshold in terms of risk for bacteria with a MIC of 2 mg/L (36, 37). In our study, for two neonates, the SCR level was higher after than before treatment, but linking the accountability to vancomycin was difficult (one neonate in a context of perinatal asphyxia and the other with multiorgan failure in a context of extreme prematurity). Studies of vancomycin-associated nephrotoxicity in neonatology conclude that the condition is rare and reversible and proof of a dose–toxicity relationship is lacking, even with levels >30 mg/L or associated with an aminoglycoside (38). However, careful monitoring should be carried out especially in high-risk neonates with nephrotoxic comedication (non-steroidal anti-inflammatory drugs, diuretics), with low birth weight or with severe illness (sepsis, patent ductus arteriosus).

CGA and high SCR level have been linked to interindividual variability in vancomycin levels, along with other covariates such as weight, PNA, GA, ventilation type, and co-treatments (4). However, residual variability remains despite accounting for these factors. Thus, therapeutic drug monitoring still seems necessary. The usual timing for vancomycin drug monitoring is at least 18 to 24 h after the initation or modification of treatment (20). Dosing adjustment was not satisfactory in our study, with 63% of levels in secondary samples remaining outside of the target. A method based on a linear relationship between the maintenance dose and steady-state level has been proposed: adjusted dose = maintenance dose × target level/last vancomycin level (19, 20).

Our study has several limitations. First, it was a retrospective study that can imply imprecision in recorded data. Second, SCR level was available before treatment for only three-quarters of the neonates, which prevented us from applying published dosing regimens to all our patients. Following this study, creatinine measurement became mandatory to manage vancomycin treatment in our department. Another limitation is the lack of documentation of MICs of bacteria, with 74% of blood cultures remaining negative. Therefore, we could not evaluate the proportion of MICs >1 mg/L that would require higher vancomycin levels. Finally, even if other studies already described the use of continuous infusion vancomycin in neonates, our cohort mainly contained extremely premature neonates <28 weeks' GA, a very challenging population.

## Conclusions

Continuous-infusion vancomycin for neonates offers a practical and pharmacokinetic interest. The use of a dosing regimen taking into account CGA and SCR level or an individualized pharmacokinetic model can help to anticipate the inter-individual variability in neonates and seems more adapted than a simplified dosing regimen. Validation of a dosing regimen in a controlled trial with a large number of patients would allow to evaluate performances in achieving target levels and for better assessment of short- and long-term toxicity. Despite a patient-tailored dosing regimen, therapeutic drug monitoring still seems necessary.

## Ethics Statement

The local ethics committee of Créteil hospital approved the anonymous collection of data and their publication.

## Author Contributions

MT and RC designed and conducted the study. MT collected and analyzed patient data. MT, RC, XD, and LC interpreted results. MT wrote the manuscript with major contributions by RC and reviewing by LC, XD, GD, and JB. All authors read and approved the final manuscript.

### Conflict of Interest Statement

The authors declare that the research was conducted in the absence of any commercial or financial relationships that could be construed as a potential conflict of interest.

## Abbreviations

CoNS: coagulase-negative staphylococci
MRSA: methicillin-resistant *Staphylococcus aureus*
GA: gestational age
CGA: corrected gestational age
PNA: post-natal age
SCR: serum creatinine
AUC_0−24h_: 24-h area under the curve
MIC: minimum inhibitory concentration
NICU: neonatal intensive care unit
IUGR: intra-uterine growth restriction
EUCAST: European Committee on Antimicrobial Susceptibility Testing.
